# Omental Flap to Non-healing Posterior Trunk Wound: A Case Report

**DOI:** 10.7759/cureus.20178

**Published:** 2021-12-05

**Authors:** Jake Laun, You Jeong Park, R. Maxwell Rotatori, Ricardo Gonzalez, Nicholas Panetta

**Affiliations:** 1 Plastic Surgery, University of South Florida, Tampa, USA; 2 Sarcoma, Moffitt Cancer Center, Tampa, USA

**Keywords:** posterior chest wall reconstruction, chronic back wound reconstruction, back reconstruction, posterior trunk reconstruction, omental flap

## Abstract

Large posterior trunk wounds often require flap reconstruction. One option for posterior truncal reconstruction not readily considered, often due to the combined anterior and posterior approaches required for harvesting and coverage, is the omental flap; however, the omental flap stands as a robust backup in non-healing wounds when local flap options have been exhausted. We present a case of a posterior trunk wound that had previously undergone multiple unsuccessful local and regional flaps for reconstruction and was ultimately treated with a pedicled omental flap which went on to heal without any post-operative complications.

## Introduction

Posterior trunk wounds pose a challenge for the reconstructive surgeon. Whether a defect from after tumor resection, trauma, or post-operative wound breakdown after spine surgery, coverage of the defect often requires flap reconstruction. Several local options exist for coverage including the latissimus dorsi flap, trapezius flap, and even free flaps depending upon the location of the defect [1]. In hard-to-heal wounds, multiple flaps may need to be performed and other options may need to be explored if there are continued issues with wound healing. One such option that has been rarely performed for posterior trunk wounds is the pedicled omental flap [2]. The omental flap is derived from the greater omentum, a large, flat layer of adipose tissue that extends from the greater curvature of the stomach to the transverse colon before reaching the posterior abdominal wall. Due to its immunological properties and rich vascularization from the gastroepiploic arteries, the greater omentum holds great potential for wound healing and has been used in diverse reconstructive scenarios [3,4]. Despite these qualities, the use of an omental flap for a posterior trunk defect has been rarely described. We present the following case as an example of a non-healing wound that was successfully treated with a pedicled omental flap.

## Case presentation

**Figure 1:**
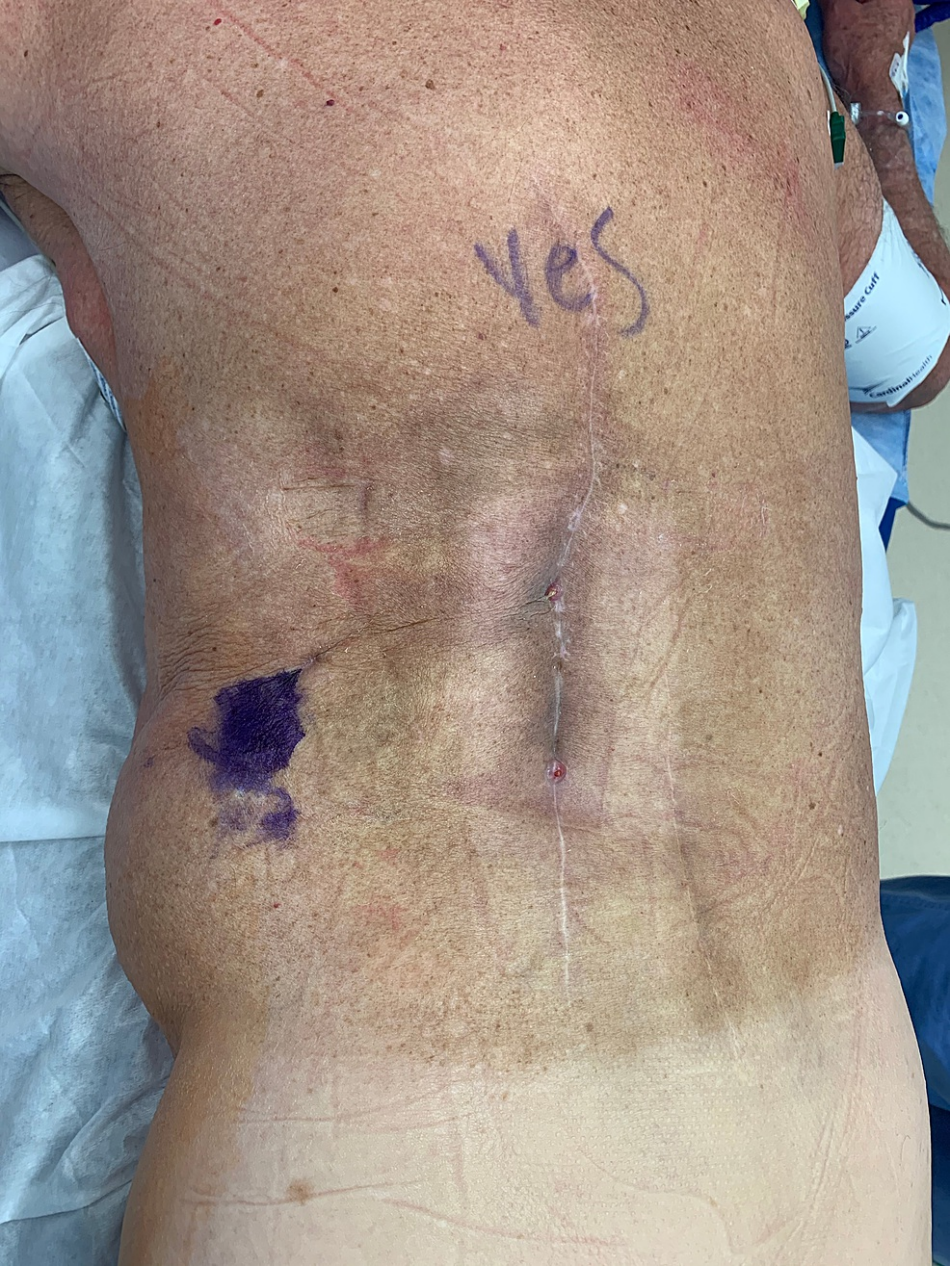
Left lower back wound showing previous incision and the chronic draining sinus in the incision. The purple marks where the mental flap was tunneled posteriorly.

Our patient is a 78-year-old man with a left lower lumbar back chronic draining wound after chemotherapy, radiation, and excision of a recurrent retroperitoneal sarcoma (Figure 1). His original excision was four years prior and, unfortunately, recurred within one year. He underwent multiple rounds of chemotherapy and radiation prior to en bloc resection of the erector spinae muscles, 12th rib, a portion of the left hemidiaphragm, and Gerota's fascia along with a portion of latissimus and oblique muscles. His original reconstruction utilized a combination of a composite latissimus dorsi, serratus anterior, and external oblique muscle flaps; however, within three months, he developed a draining sinus tract that was eventually excised and reconstructed with advancement of a left back fasciocutaneous flap (with quilting and progressive tension suture placement). Unfortunately, the drainage recurred again and a CT scan demonstrated evidence of reaccumulation of cystic fibrinous tissue. The decision was made to bring in healthy, well-vascularized tissue to mitigate the radiation damage that he had incurred and to augment healing. Given the lack of other loco-regional options, an omental flap was chosen. 

**Figure 2:**
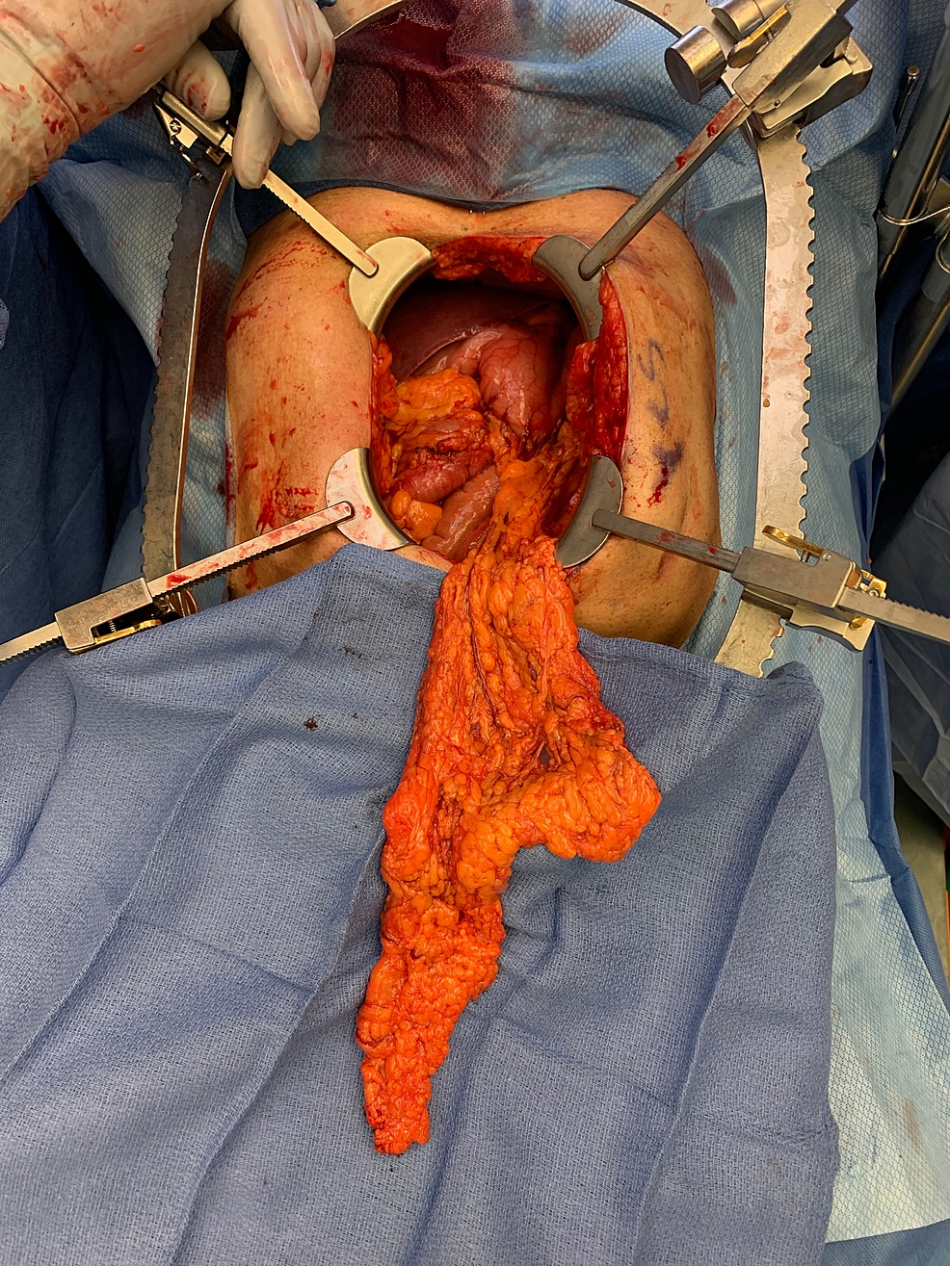
Omental flap harvest from the anterior approach showing the mental flap still attached to the left gastroepiploic vessels.

**Figure 3:**
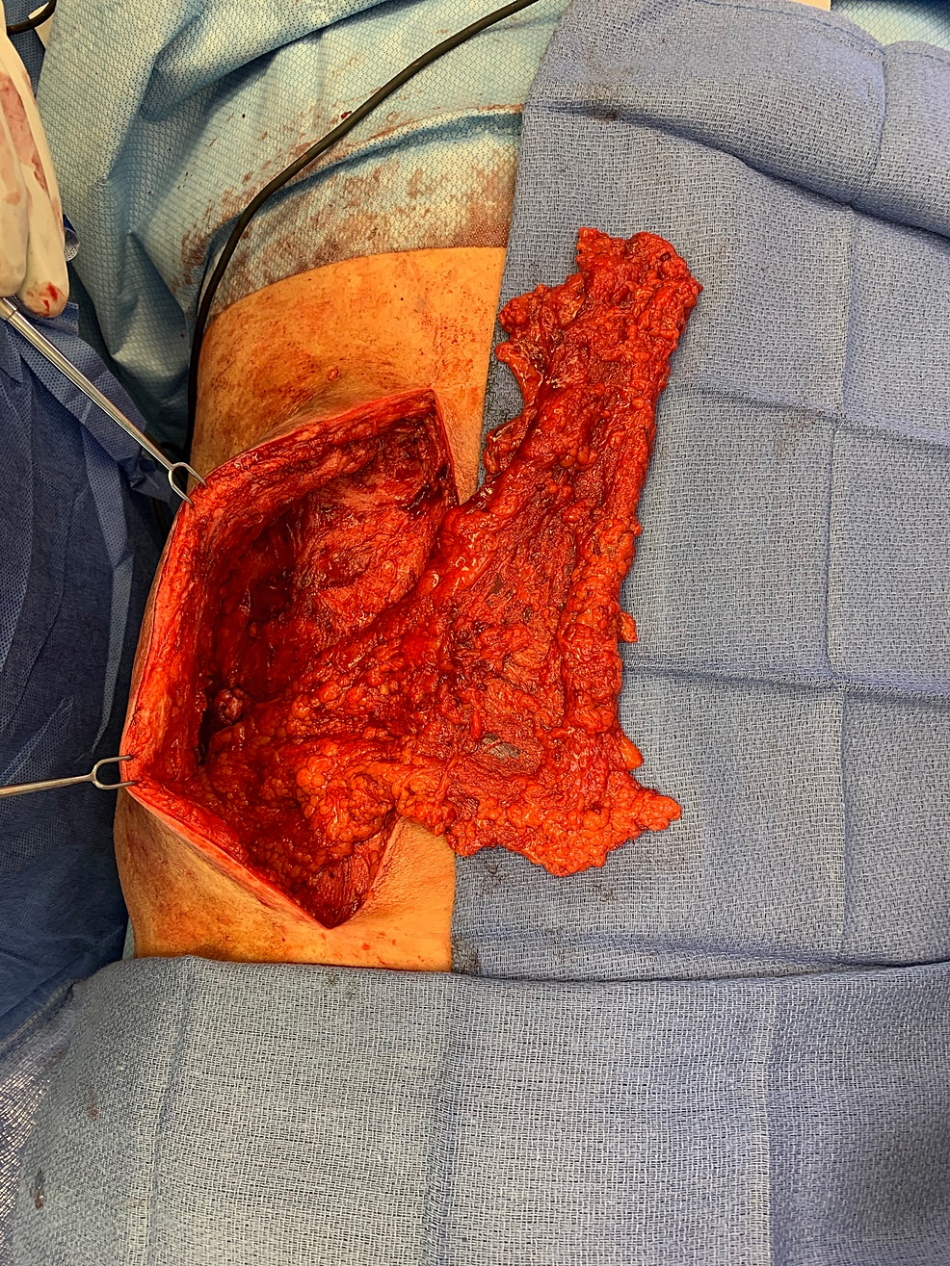
Omental flap transposed through retroperitoneum and brought out posteriorly into the chronic wound after the wound was debrided of all necrotic and radiated tissues.

**Figure 4:**
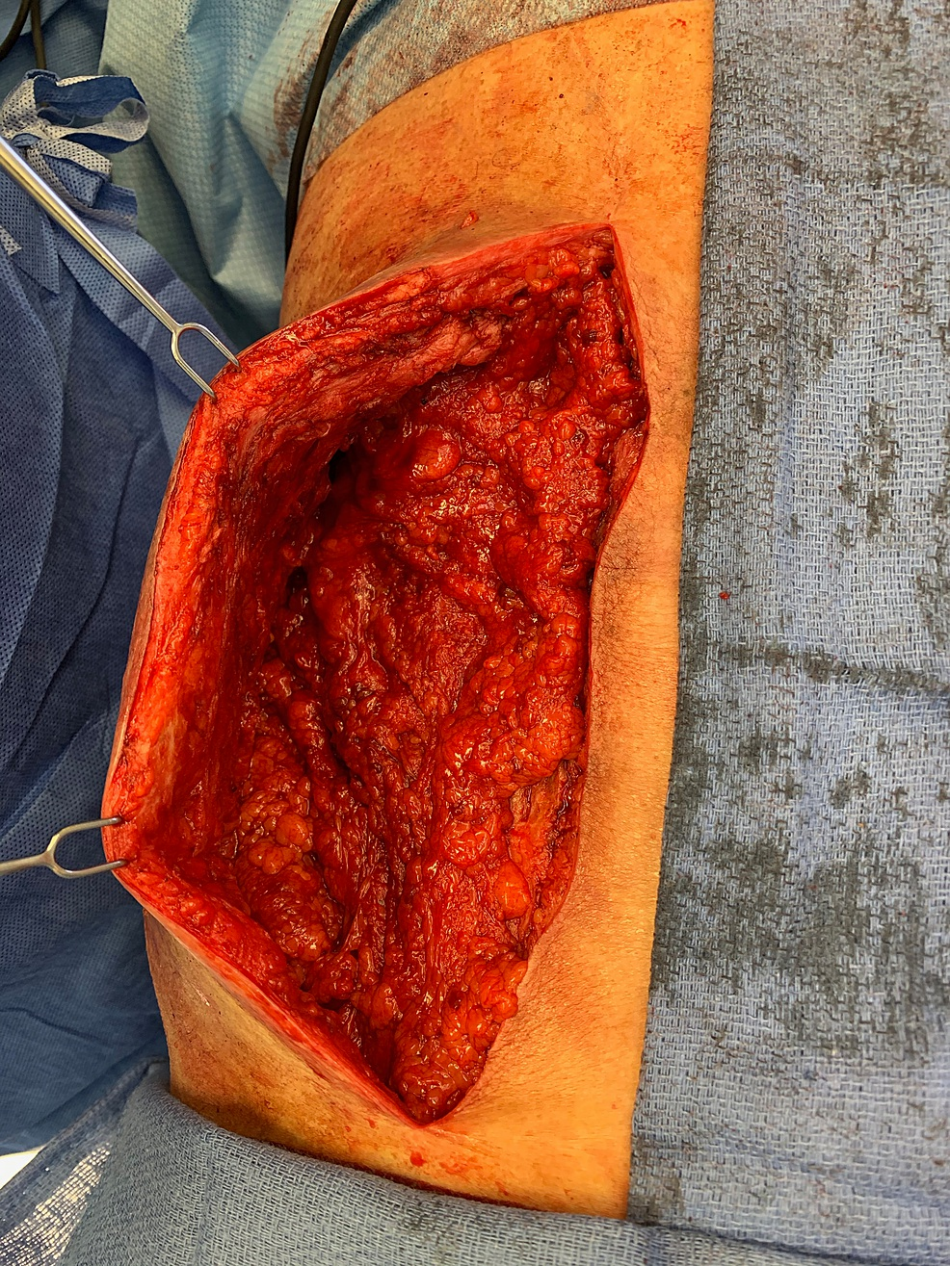
Omental flap was then draped and laid out into the wound to provide an obliteration of the dead space as well as bring in the healthy, vascularized tissue to the wound.

**Figure 5:**
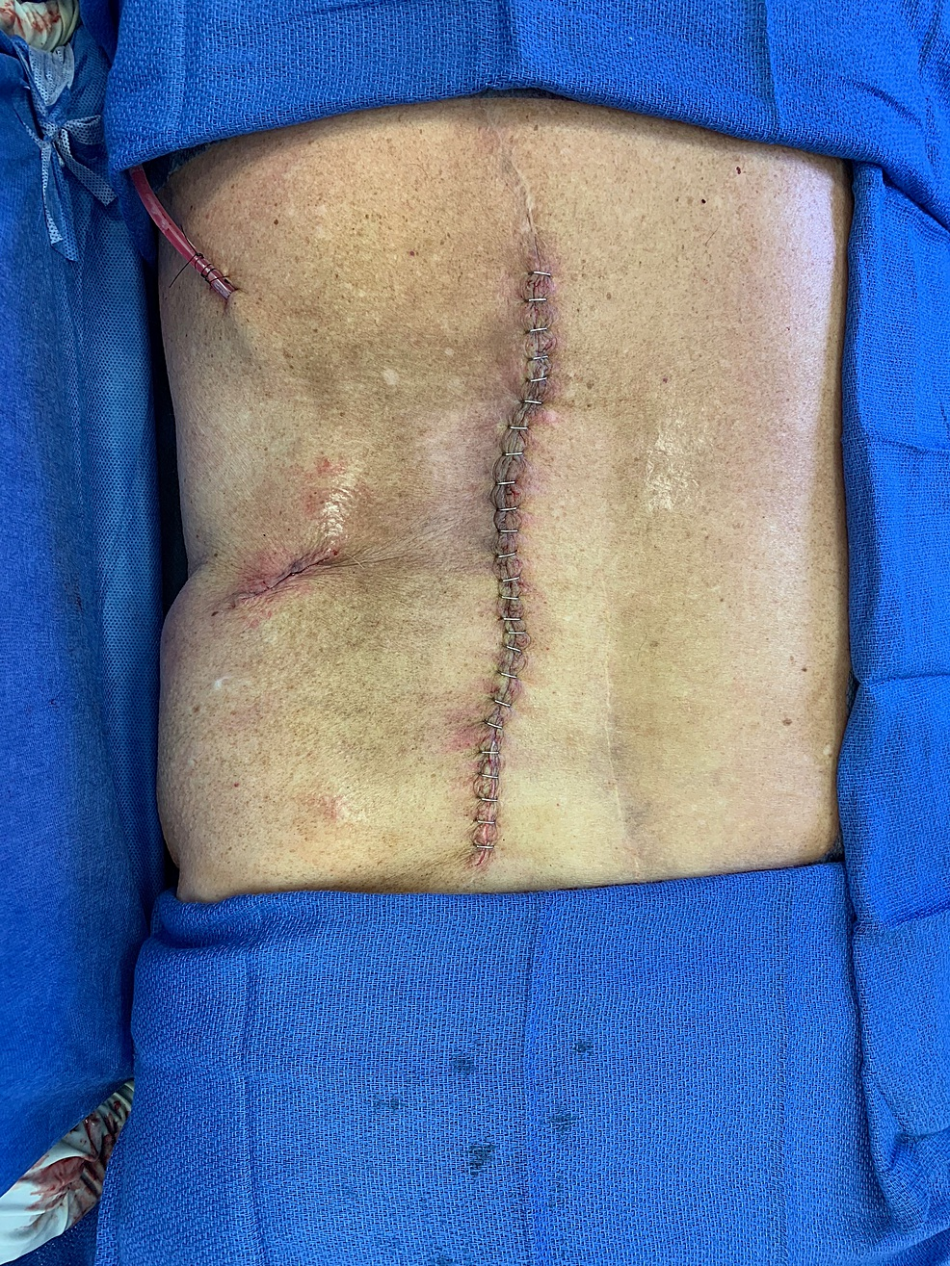
Final closure after the sinus track and the necrotic/radiated tissue was excised and the mental flap was draped into the wound. A JP drain was placed into the wound and the overlying skin flaps were brought together there and closed.

With the assistance of the general surgery team, the omental flap was harvested via a supra-umbilical midline laparotomy. The omentum was mobilized off of the transverse colon and based on the left gastroepiploic vessel (Figure 2). The left transverse colon was mobilized off the retroperitoneum and medialized to a point where the flap could be transposed through into the back directly. Harvesting of the flap as well as the transfer of the flap into the retroperitoneal space should be carefully performed in order to avoid any injury to the colon or other retroperitoneal structures. Palpation of this retroperitoneal location was marked externally on the skin of the back. A 1-inch Penrose drain was sutured to the distal end of the omentum to assist in transfer of the flap through the retroperitoneum. After the midline was closed, the patient was placed in a prone position and the back wound underwent excisional debridement of radiated tissues with the final defect measuring 15 x 20 cm. The retroperitoneum was accessed through a small opening in the base of the wound and the omental flap was identified and transposed into the back wound (Figure 3). After inset of the flap, the omentum had good Dopplerable signals and was draped throughout the posterior back wound cavity (Figure 4). Subsequently, a layered closure of the back over the top of the omentum was performed and a subcutaneous drain was placed (Figure 5). Postoperatively, the patient was positioned in a manner to avoid compression of his left back. The rest of his hospital course was uneventful and he was discharged on post-operative day 5 after return of bowel function and his drain was removed at three weeks. His post-operative course has been uneventful to date and was just seen now over two years post-operatively with no recurrence of his wound.

## Discussion

Whether as a result of a congenital defect, trauma, oncologic resection, or pressure sore, posterior trunk wounds often require closure using a flap since skin grafts fail to provide sufficient long-term stability and durability. Flaps typically used for posterior trunk reconstruction include the trapezius, latissimus dorsi, and gluteus maximus muscles [1,5]. Secondary flap options include the scapular, parascapular, paraspinous, and intercostal muscles [1]. Selection depends on the size and location of the defect and the anatomy and arc of rotation of available flaps. 

Despite its versatility, the pedicled omental flap is infrequently used for posterior trunk wounds because harvesting requires a laparotomy and introduces the risk for intra-abdominal complications such as infections, fascial dehiscence, hernia, and post-operative ileus [6]. However, in cases where local flaps are unavailable or free and local flaps fail, the omental flap stands as an excellent alternative for coverage due to its large surface area, vascularity, malleability, and generous pedicle length. In our patient’s case, due to multiple prior reconstructive attempts and in setting of radiation damage to the local soft tissue, few options remained. An omental pedicle flap based off the left gastroepiploic artery thread through the left paracolic gutter provided sufficient length and bulk to reach and fill the defect and be subsequently buried under local skin flaps. 

A few considerations should be made when performing an omental flap for posterior trunk defects. Harvesting the omentum based off the left gastroepiploic artery is hypothesized to pose less risk for splenic and renal injury than the right [7]. Postoperatively, the patient should be positioned to avoid compression of the lower back. Finally, factors associated with donor-site complications can include mediastinitis, advanced age, and pulmonary failure [6].

## Conclusions

Reconstruction of the posterior trunk presents a complex and challenging problem to the reconstructive surgeon. Local tissue rearrangement, regional flaps, or even free flaps can be used to close these defects, which can be even more challenging when in the setting of radiation. In cases such as ours, when local options have been utilized and other regional flaps have also been exhausted, the omental flap can provide a valuable and well-vascularized pedicled flap of tissue that can be mobilized and transferred into a non-healing wound to assist with healing by providing increased vascularization into the chronic wound bed. We present a case utilizing a pedicled omental flap where the patient was able to ultimately heal a chronic posterior trunk wound that was complicated by the previous utilization of local and regional flap options.
